# Mammalian Stem Cells Reprogramming in Response to Terahertz Radiation

**DOI:** 10.1371/journal.pone.0015806

**Published:** 2010-12-31

**Authors:** Jonathan Bock, Yayoi Fukuyo, Sona Kang, M. Lisa Phipps, Ludmil B. Alexandrov, Kim Ø. Rasmussen, Alan R. Bishop, Evan D. Rosen, Jennifer S. Martinez, Hou-Tong Chen, George Rodriguez, Boian S. Alexandrov, Anny Usheva

**Affiliations:** 1 Department of Medicine, Endocrinology, Harvard Medical School, Beth Israel Deaconess Medical Center, Boston, Massachusetts, United States of America; 2 Center for Integrated Nanotechnologies, Los Alamos National Laboratory, Los Alamos, New Mexico, United States of America; 3 Theoretical Division, Los Alamos National Laboratory, Los Alamos, New Mexico, United States of America; Centro Cardiologico Monzino, Italy

## Abstract

We report that extended exposure to broad-spectrum terahertz radiation results in specific changes in cellular functions that are closely related to DNA-directed gene transcription. Our gene chip survey of gene expression shows that whereas 89% of the protein coding genes in mouse stem cells do not respond to the applied terahertz radiation, certain genes are activated, while other are repressed. RT-PCR experiments with selected gene probes corresponding to transcripts in the three groups of genes detail the gene specific effect. The response was not only gene specific but also irradiation conditions dependent. Our findings suggest that the applied terahertz irradiation accelerates cell differentiation toward adipose phenotype by activating the transcription factor peroxisome proliferator-activated receptor gamma *(PPARG)*. Finally, our molecular dynamics computer simulations indicate that the local breathing dynamics of the PPARG promoter DNA coincides with the gene specific response to the THz radiation. We propose that THz radiation is a potential tool for cellular reprogramming.

## Introduction

Terahertz (THz) radiation occurs ubiquitously in our environment, as part of the solar spectrum and through the natural black-body radiation within the earth's atmosphere. Despite this abundance, the non-ionizing character of this radiation, and the lack of practical and powerful THz emitters, has left the biological significance of this region of the electromagnetic spectrum relatively unexplored. Interestingly, the energy scale of THz radiation is within the range of hydrogen bonds, van-der-Waals interactions, and charge-transfer reactions. This energy overlap is associated with the unique sensitivity of the emerging THz techniques [1], [2] to the molecular motions that underlie intricate biological functions. These distinctive properties, together with the nascent development of powerful THz sources [1] and the resulting broad spectrum of applications [3], now pose optimal conditions for understanding the nature of the interactions between THz radiation and biomolecules.

Unfortunately, the available data related to the influence of THz radiation on biological systems, and the understanding of the precise mechanisms governing this influence, are limited and the subject of debate [4]. Previous *in vitro* and *in vivo* studies were mostly conducted at frequencies below 0.15 THz, at low power, and with short exposure times (10–30 min), and did not provide conclusive evidence regarding the influence of THz radiation on mammalian cells. Further, results from the multi-national “THz-bridge” project aimed at investigating the interaction of THz radiation with biological systems [5], reported potential genotoxic and epigenetic effects on human lymphocytes and changes in the membrane permeability of liposomes, but most critically was unable to clarify the exact irradiation conditions necessary to cause these effects. More recent studies confirm that a weak THz field may cause genomic instability in human lymphocytes [6] after extended (6 hours) exposure. Likewise, it was reported that neurons briefly exposed *in vitro* to powerful THz radiation (over 30 mW/cm^2^), at a specific frequency, develop infringements on the morphology of the cellular membranes and intracellular structures [7]. Finally, changes in the gene expression have been documented after prolonged exposure (72 hours) to low-power broad-spectrum THz radiation centered at ∼30 THz [8]. Importantly, all these experiments were conducted under controlled thermal conditions to ensure that temperature is unrelated to the observed effects. It thus appears that THz radiation can interfere with biological functions in genomic materials. While, it remains unclear whether THz radiation is influencing specific genomic functions or whether the impact is more general resulting in cellular damage, it is apparent that the mechanisms by which the non-ionizing THz radiation influences biological functions must be fundamentally different from those at play when high-energy (UV, x-ray, gamma, etc.) radiation interacts with bio-matter. Indeed, prior research [9]–[10] provides ample evidence that exposure to THz radiation can affect intramolecular vibrations and hence dynamically induce new conformational states of proteins [11]. These new conformations may easily perturb, for example, protein-DNA binding and thereby induce changes in cellular transcription and replication.

Here we report that extended exposure to broad-spectrum THz radiation (centered at ∼10 THz) results in specific (rather than global) changes in the functionality of cellular DNA. Certain genes in irradiated mouse stem cell (MSC) cultures are activated, while other genes are repressed. Many of the MSC genes do not respond to the selected radiation conditions at all, showing that the effect is specific. Additionally, 9 hours of exposure causes significant changes in the MSC gene expression, while the response to shorter duration (2 and 4 hours) is appreciably less pronounced. Hence, we argue that the effect of THz radiation is gene and exposure specific and most likely is at the level of DNA transcription. In this context, our EPBD-based [12], [13] Langevin computer simulation modeling shows that the promoter DNA propensity for local breathing is likely to be one of the factors that underlie the gene specific response to the applied conditions of irradiation. In light of these data, together with our previous demonstration of strong correlations between transcription and the propensity of dsDNA for local breathing [12]–[17], it is natural to consider the influence of nonlinear THz resonances upon DNA breathing dynamics [18] as one of the factors in the cellular response to THz radiation.

## Results

### Morphological changes in mouse stem cells after THz irradiation

**Figure 1:**
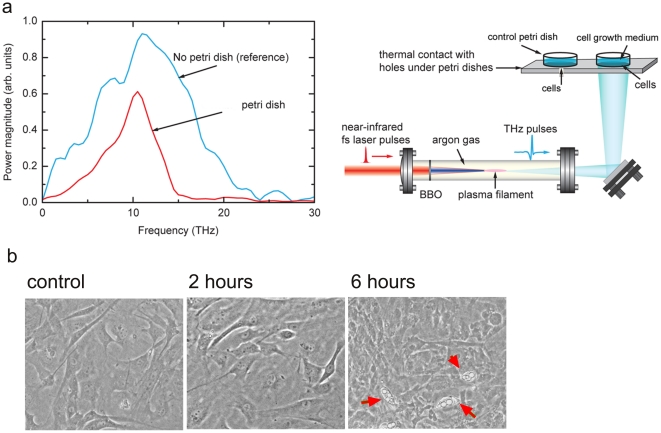
THz irradiation of mouse stem cells. a) THz radiation was generated by a frequency-doubling BBO crystal, in Argon at 600 torr pressure, with 1 KHz repetition rate [31]. The irradiated and control cells were in thermal contact and the temperature of both samples was monitored by themosensors. b) Mouse stem cells were monitored by light microscopy for morphological changes in response to THz exposure. A significant accumulation of lipid-like droplets in the cellular cytoplasm was visible in response to 6 hours of THz irradiation. Representative photographs are shown with 100x magnification for: Cells without THz exposure; cells after 2 hours of THz exposure; and cells after 6 hours THz exposure. Cells with an increased number of lipid-like droplets inclusions in the cytosol are indicated with black arrow; orange arrows – undifferentiated cells; white arrows – initial stage of adipogenesis.

It was previously shown that intensive THz irradiation results in changes of neuronal cellular membranes that can be monitored by light microscopy [7]. We exposed MSC cultures to a broad-spectrum (centered at ∼10 THz) THz radiation (Figure 1, a) for 2 and 6 hours at an average power density of ∼1 mW/cm^2^. To exclude any external variations in the growth conditions, unrelated to the THz radiation, two MSC culture dishes, control and experimental, were placed next to each other during the THz treatment (Figure 1, a) and further processed for analysis in parallel. For the irradiated sample, there was no observed effect on the cell membranes. However, lipid droplet-like inclusions appeared in the cellular cytoplasm after 6 hours of exposure (Figure 1, b). Significantly, fewer cells were observed to contain such inclusions in the cells exposed only for 2 hours and in the control MSC cultures, suggesting that the morphological changes observed in the MSC cytoplasm are specific for the selected duration of exposure.

### Effect of THz irradiation on gene expression in mouse stem cell cultures

**Figure 2:**
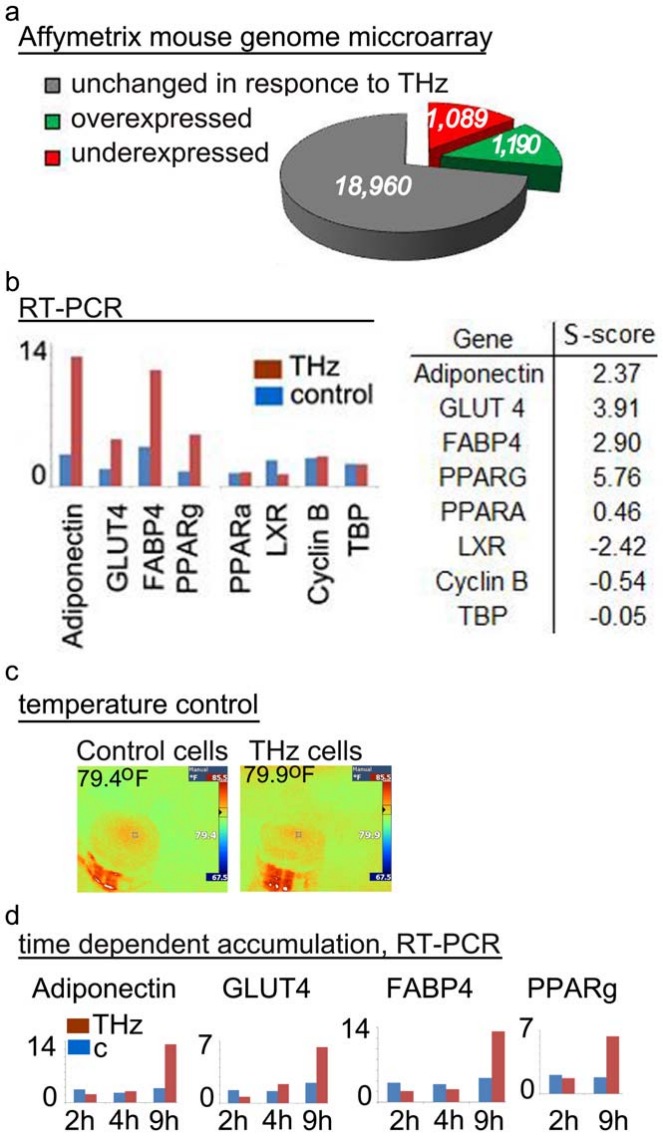
Gene specific effect of THz irradiation in mouse stem cell cultures. a) Gene expression profiling of MSC cells in response to 9 hours of THz exposure. Of ∼21,000 annotated genes represented in the Affymetrix mouse genome microarray, 1050 were underexpressed (red) and 1154 were overexpressed (green) with statistical significance p<0.05 as compared to the non-irradiated parallel control; b) RT-PCR measurement for selected genes. The relative level of gene expression in response to 9 hours exposure of MSC to THz radiation normalized to the TBP gene. The identity of the genes is shown below the bars. Cells without THz treatment served as control. The experimental results are consistent between three independent RT-PCR measurements in duplicates and in two different sets of irradiation. Brown bars – THz irradiated cells; blue bars – control cells. The table lists the S-Scores (representing the change in expression level in response to THz irradiation compared to the nonirradiated control) for these genes derived from the Affymetrix mouse genome microarray. c) The temperature was monitored using an IR detector, and separately using thermo-sensors glued to the outside of the petri dish lids. The temperature at the end of irradiation for the control plate with cells (c-79.4^0^F) and the THz irradiated cells (THz-79.9^0^F) is shown above the snap shots panels (snap shots are from the IR detectors in Fahrenheit). d) RT-PCR measurements for selected genes in response to 2, 4, and 9 hours irradiation. RNA levels are normalized to the TBP gene. The identity of the gene is shown at the top. The duration of irradiation is shown below the bars in hours (h). The number of specific transcripts is shown on the vertical axis in relative units [R.U.]. The RT-PCR results are consistent between two independent sets of measurements in duplicates.

Changes of specific gene expression under prolonged exposure to a very low power broad-spectrum THz radiation (centered at ∼30 THz) have been reported in an experiment with the eukaryotic microorganism *Tetrahymena thermophila* [8]. Hence, it appears likely that THz irradiation has a direct effect on cellular levels of RNA transcripts. The question arises of whether mammalian cells will respond to THz irradiation at the level of DNA gene transcription. Therefore, following 9 hours of exposure to broad-spectrum THz radiation (Figure 1a), MSCs were collected and analyzed for changes in global gene expression. We compared the level of RNA transcripts in irradiated and control cells in a set of hybridization analyses with a gene array containing probes for over 21,000 well-characterized and annotated protein coding mouse genes. Our data shows that the genes in the irradiated cells can be categorized into three different groups with regard to the changes in RNA transcript content: 1) genes with an increased level of RNA transcripts, 2) genes with a reduced level of RNA transcripts, and 3) genes that remain with the same RNA transcript level when compared to the non-irradiated control cells (Figure 2, a). Specifically, as a result of the irradiation, ∼6% of the genes on the array were found to increase (more than two times) the level of RNA transcripts, ∼5% decreased (more than two times), and ∼89% remained the same as in the control cells. *This data is the first body of evidence that the exposure of mammalian stem cells to THz radiation results in transcriptional gene specific changes in expression*.

To validate the array data, namely that the response to the radiation is gene specific, we measured quantitatively the messenger RNA (mRNA) levels using RT-PCR. With selected available gene probes, corresponding to transcripts from the three groups of genes identified in the array experiment, we confirmed the observed gene array results (Figure 2, b). Four of the selected clones, that is: the transcription factor PPARG [19], adiponectin [20], GLUT 4 [21], and FABP4 [22], were confirmed to be present at more than two times higher level in the irradiated cells relative to the control cells. Decreased levels of mRNA transcripts in irradiated MSC were confirmed for the nuclear receptor transcription factor LXR [23]. No measurable changes were observed for the transcription factor alpha PPARA [24], TATA binding protein (TBP) [25], and cyclin B [26]. These observed responses are directly related to the THz radiation and not to changes of temperature, because the temperature in the control and the experimental cell culture dishes remained nearly identical (79.7°F+/−0.3°F), as shown in Figure 2, panel c.

The observed accumulation of mRNA depends on the duration of exposure. The accumulation of adiponectin, GLUT 4, FABP4 and PPARG mRNA transcripts was evaluated by RT-PCR after 2, 4, and 9 hours irradiation. The results for the selected time frames revealed clear difference in the accumulation of specific mRNA transcripts (panel d). Significant changes in specific mRNA transcripts levels were measured only after 9 hours of exposure. The level of TBP transcripts did not change detectably and was used as an internal control for equal sample loading.

### Correlation between the gene specific intrinsic dsDNA breathing dynamics and the transcriptional MSC response to THz irradiation

**Figure 3:**
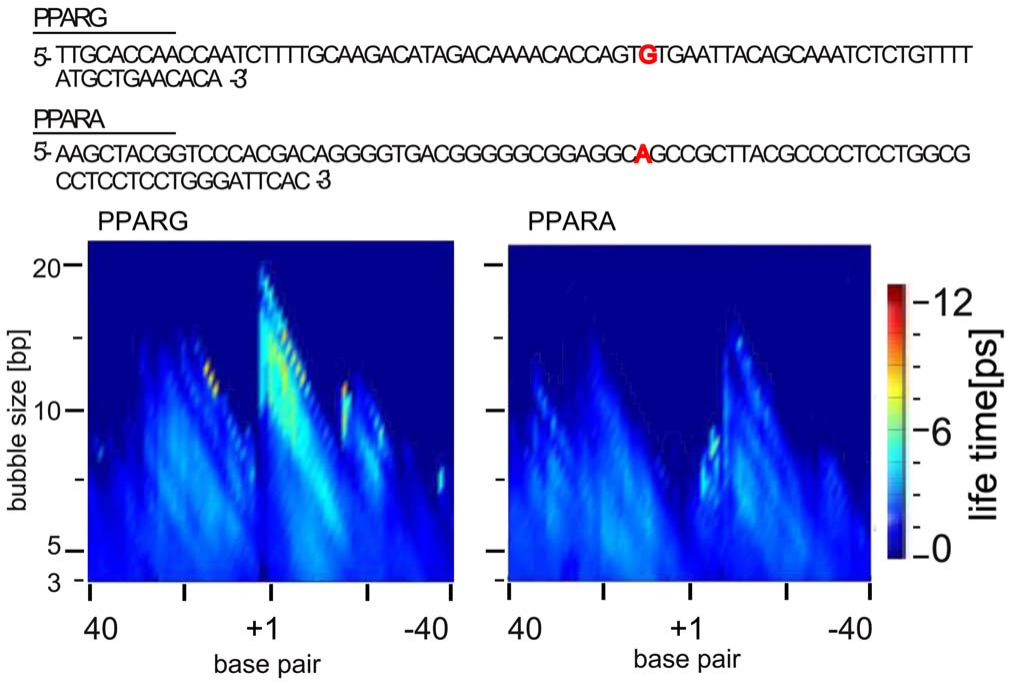
Computer simulation data. EPBD based Langevin molecular dynamics showing the intrinsic breathing dynamics in the promoter regions of PPARG and PPARA genes: vertical axis - size of bubbles [base pairs, bp] with defined average lifetimes; horizontal axis - nucleotide position [base pair] where the TSS is labeled ‘+1’. The color bar on the right indicates the bubble lifetimes in pico seconds [ps]. The promoter sequences obtained from the DBTSS (http://dbtss.hgc.jp), are shown above the simulation data panel. The red letter indicates the TSS position. The identity of the gene sequence is shown above the plots.

PPARG and PPARA belong to the same group of nuclear receptor proteins, with overlapping activity as transcription factors, and a well-documented difference of transcriptional regulation of their DNA gene promoters [19], [24]. Interestingly, the response of these genes to the applied irradiation conditions is quite different: PPARG is transcriptionally activated, while PPARA does not respond even in the case of 9 hours of exposure (Figure 2, b). Computer simulation data obtained by our EPBD Langevin molecular dynamics shows that these two promoters differ substantially in their intrinsic breathing dynamics at the transcription start site (TSS) regions (Figure 3). The PPARG promoter demonstrates the characteristic dynamic pattern previously observed for other mammalian promoters, [13], [15] characterized by a major site of dynamical activity in close proximity to the TSS. In contrast, the simulations of the PPARA promoter show a dynamic activity that is almost evenly distributed and noticeably weaker than that of PPARG: PPARA is clearly lacking the characteristic long-lived bubbles at the TSS observed for PPARG. Importantly, this difference in the local TSS breathing dynamics coincides closely with the observed response to the THz irradiation.

## Discussion

THz radiation, with a fundamental period in the pico-second range, is uniquely suited to control functions in molecular systems of central importance for living organisms. Biologically important collective modes of proteins vibrate at THz frequencies and are therefore THz radiation sensitive [27]. The hydrogen bonds in dsDNA also vibrate at a THz frequency, and THz radiation has the potential to change important cellular genomic DNA and DNA-protein functions. Although the THz photons do not carry enough energy to directly alter chemical reactions, nonlinear resonance effect may cause local changes of the breathing dynamics in systems such as DNA [18], leading to changes in gene transcription.

Here we report that extended exposure to a broad-spectrum THz radiation results in specific rather than global changes in cellular functions that are closely related to the DNA-directed gene transcription. Our gene chip and RT-PCR survey on gene expression reveals that some genes in irradiated MSC cultures are activated, while other genes are repressed. Yet other genes do not respond to the selected radiation conditions at all, demonstrating the gene specific, rather than general, effect on gene transcription. Our experimental data further argues that the response is not only gene specific but also depends on the conditions of irradiation, as cell morphology and gene specific accumulation of RNA transcripts was dependent on exposure duration. Further, it is known that THz radiation affects biomolecules via interaction with the internal dipole moments [28]. The previous experimental findings [29] that gene promoter and non-promoter sequences possess significantly different dipole moments are consistent with our present observations that THz radiation interacts in a sequence-specific manner with DNA.

Exposure of cells to THz radiation for 9 hours causes changes in gene expression, while in response to shorter duration of exposure the changes are significantly less pronounced. The appearance of lipid droplet-like inclusions that is a characteristic sign of MSC differentiation into adipocytes, was clearly visible after 9 hours exposure rather than in the typical time frame of several days. This may be explained by the observed significant elevation of the PPARG expression. This transcription factor is known to be required for transcriptional activation and expression of adipocyte genes including adiponectin and GLUT4 that produce the differentiated phenotype [30]. Our finding suggests that THz radiation with the selected parameters enhances the differentiation process towards this adipocyte-like phenotype in MSC.

The observations of the variations of DNA transcription due to THz radiation support a correlation between specific THz radiation effects, the level of accumulation of gene specific RNA transcripts, and the natural nonlinear process of DNA transcription initiation. One particularly pronounced variation in gene transcription is between PPARA and PPARG. Our EPBD based Langevin molecular dynamic simulations show that the DNA propensity for local breathing at the PPARG promoter is significantly higher, and focused at the TSS compared to the noticeably dynamically silent PPARA. The correlation with the THz induced PPARG promoter activation is striking. This difference indicates that the selected irradiation parameters, and the breathing promoter signature, may together direct the functional response to THz irradiation. On the other hand a PPARA response may require an extended exposure, since DNA breathing with higher amplitudes is needed for the THz effect to take place via nonlinear resonances [18]. The absence of extended exposure may also underlie the absence of transcriptional response for the selected gene examples after 2 hours of irradiation. It is also likely that, for certain genes, possibly including PPARA and TBP, transcription depends solely on deterministic factors such as protein binding, while genes such as PPARG could mainly depend on probabilistic factors such as DNA breathing dynamics. It is likely that the difference in the promoter specific breathing dynamics contributes to the observed response to THz irradiation [18], and therefore may serve to further extend our understanding of the observed difference in the gene specific response including PPARA and PPARG.

In addition to effects pertaining to the formation of DNA transient openings, it is also possible that DNA-protein interactions and transcription factor conformational changes could be induced by THz irradiation [11]. Such interactions could result in favorable transcription or cause interference with transcription. In the nuclear receptor transcription factor LXR, RNA transcript levels were reduced and may be explained through DNA-protein interactions. In either case, the data shows that the effect of THz radiation conditions is gene specific and most likely at the level of DNA transcription. Our EPBD Langevin molecular dynamic simulations indicate that the promoter DNA propensity for local breathing may be an important factor in the gene specific response to the selected conditions for irradiation.

Further investigations involving a large number of genes and variations in the THz radiation characteristics as well as longer time exposure are needed to generalize these striking correlations between promoter-specific breathing dynamics, probability for THz induced nonlinear resonances, transcriptional response, and cellular behavior.

## Materials and Methods

### Mouse stem cell culture

Mouse mesenchymal stem cells (ScienCell Research Laboratories, CA 92011, Catalog Number M7500) were cultured on tissue-culture treated plastic T-75 flasks (Corning). Once the cells reached 80–90% confluence, they were sub-cultured in supplemented medium (95% α-MEM, 5% FBS, and antibiotics) for THz irradiation treatment. Roziglitazone (1 µM), insulin (5 µg/ml), 3-Isobutyl-1-methylxanthine (100 µM), and dexamethasone (1 µM) were added to the medium 24 hours before irradiation.

### THz irradiation

Through the frequency mixing of ultrafast fundamental and second-harmonic laser fields, a directional plasma electron current in pressurized atomic gases can be generated. In case of ultrafast lasers (<100 fs) this technique is capable of producing electromagnetic radiation at THz frequencies [31]. We use a recently developed coherent-control scheme optimizing for such type of THz generation in gases, yielding a new source of high-energy (∼1 µJ, pulse width 35 fs i.e. high peak power per pulse ∼30 MW), average density power 1 mW/cm^2^, broadband THz radiation (∼10 THz) at a high repetition rate (1 kHz). Cells were irradiated in parafilm-sealed tissue culture plates in the presence of culture medium and at ambient temperature. Cells were irradiated for 2, 4, 6, and 9 hours in two replicates. Nonirradiated identical plates were used as a control in all experiments. Cell viability and morphology were monitored by light microscopy, 100x magnification (Nikon). Temperature of the cells during irradiation was monitored using an IR detector (Fig. 2), and separately using thermo-sensors glued to the outside of the petri dish lids.

### Computer simulations

Our EPBD model is an extension of the classical Peyrard-Bishop-Dauxois nonlinear model [32] to include inhomogeneous stacking potentials [12]. Our EPBD-based Langevin molecular dynamics computer simulations and the analysis of the dynamic trajectories, from which we derived the average bubble lifetimes, are performed as previously reported [15].

### Isolation of RNA and RT-PCR

Cellular RNA was extracted using the RNeasy Mini kit (Qiagen) following the manufacturer's instructions. For RT–PCR experiments, 600 ng total RNA was reverse transcribed using a cDNA Synthesis kit (Invitrogen) with random-oligo primers. For cellular qRT–PCR, 500 ng total RNA was reverse transcribed using the same conditions as above; 1/10 dilutions were used in triplicates with 0.2 µM gene specific primers and 5 µl LightCycler 480 SYBR Green I Master kit (Stratagene) in 20 µl reactions. qPCR was executed in a 96-well block Stratagene RTQPCR Mx3000 instrument. Gene specific primers were selected (PRIMER BLAST, NIH), synthesized, HPLC, and gel purified. The sequences of the primers have been published elsewhere [33]. The relative level of gene expression was normalized to the TBP gene.

### Affymetrix Gene Chip assay

A gene chip analysis assay was performed with a mixture of total RNA from cells that were irradiated for 9 hours in two independent experiments and the same procedure was performed separately for the control cells. The Affymetrix gene chip (GeneChip Mouse Genome 430 2.0 Array) hybridization procedure was conducted as recommended by the supplier (Affymetrix). The microarray data discussed in this publication have been deposited in NCBI's Gene Expression Omnibus [34] and are accessible through GEO Series accession number GSE23888.

### Data analysis and statistical methods

The comparative CT method (Applied Biosystem) was used to analyze the data resulting from the RT-PCR experiments and the gene chip analysis. Statistically significant differentially expressed genes in the Affymetrix GeneChip® microarray data were identified via the Bioconductor software framework (http://www.bioconductor.org) and the Significance Score (S-Score) algorithm [35]. S-Score was used due to the high sensitivity and non-reduced specificity for detecting low-level gene expression changes [36]. A p-value threshold of 0.05 was applied for determining statistical significance.
